# Two new species of the genus *Symphylella* (Symphyla, Scolopendrellidae) from East China

**DOI:** 10.3897/zookeys.1003.60210

**Published:** 2020-12-14

**Authors:** Ya-Li Jin, Yun Bu

**Affiliations:** 1 Natural History Research Center, Shanghai Natural History Museum, Shanghai Science & Technology Museum, Shanghai, 200041, China Shanghai Science & Technology Museum Shanghai China

**Keywords:** Chaetotaxy, morphology, Myriapoda, taxonomy

## Abstract

*Symphylella minuta***sp. nov.** and *Symphylella communa***sp. nov.** from China are described and illustrated. *Symphylella minuta***sp. nov.** is characterized by the delicate and minute body, a well-developed and thin central rod with a vestige of a transverse suture in the middle, eight setae on the first tergite, pointed processes on the tergites, and short cerci with sparse setae. *Symphylella communa***sp. nov.** is characterized by the chaetotaxy of the first tergite with 4+4 setae, processes of the tergites somewhat longer or the same length with broad, most of lateromaginal setae long, anterolateral setae of tergites 2–4, 6, 7, 9, and 10 distinctly longer than other lateromarginal setae, approximately as long as the process of the same tergite, and cerci with numerous subequal and slightly curved setae. In addition, the chaetotaxic variation on the tergites, the distribution, the habitat, and the feeding habit of the genus *Symphylella* are discussed.

## Introduction

Symphyla is an ignored group of myriapods with about 200 species reported in the world and only six species are known from China (Bu and Jin 2018; Jin and Bu 2018, 2019; Jin et al. 2019). The genus *Symphylella* Silvestri, 1902 is a common group of symphylans in soil and litter, with 49 cosmopolitan species recorded (Szucsich and Scheller 2011; Bu and Jin 2018; Jin et al. 2019). However, the study of the diversity of this group in China is still insufficient, with only two species recorded so far, *S. macropora* Jin & Bu, 2019 and *S. zhongi* Jin & Bu, 2019, both from Tibet (Jin et al. 2019). From 2012 to 2020, the symphylans in East China were comprehensively investigated in several projects and plenty of specimens were obtained. After careful identification, two new species of the genus *Symphylella* were distinguished and are described in this paper.

## Materials and methods

Specimens were collected using Berlese-Tullgren funnels and preserved in 80% ethanol. They were mounted under slides using Hoyer’s solution and dried in an oven at 50 °C. Observations were performed under a phase contrast microscope (Leica DM 2500). Photographs were taken with a digital camera installed on the microscope (Leica DMC 4500). Line drawings were done using a drawing tube. All specimens are deposited in the collections of Shanghai Natural History Museum (**SNHM**), Shanghai, China.

## Results

### Taxonomy

**Family Scolopendrellidae Bagnall, 1913**

#### Genus Symphylella

Taxon classificationAnimaliaScolopendrellidaScolopendrellidae

Silvestri, 1902

D2A18801-CE40-59FA-8CC9-BAFA507EBF47

##### Type species.

*Symphylella isabellae* (Grassi, 1886), described from Italy.

#### Symphylella minuta

Taxon classificationAnimaliaScolopendrellidaScolopendrellidae

Jin & Bu sp. nov.

31922237-5974-5D01-B840-73E2B8286D3E

http://zoobank.org/D821598B-0D68-4ABF-8906-8ED56C0CD581

**Figure 1.**
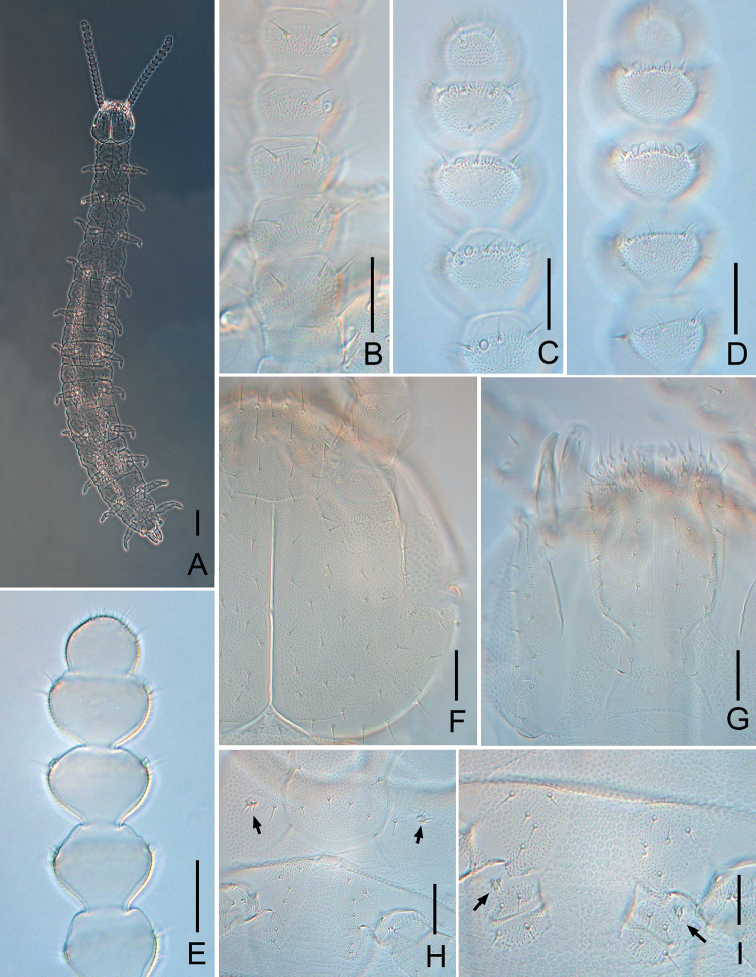
*Symphylella minuta* sp. nov. **A** habitus **B** left antenna, 1^st^–6^th^ antennomeres, dorsal view **C** distal five antennomeres, dorsal view **D** distal five antennomeres, ventral view **E** distal five antennomeres, show lateral organs **F** head, right side, dorsal view **G** head, ventral view **H** first pair of legs (arrows indicate the reduced legs) and coxa of leg 2 **I** styli, and coxal sacs on base of 3^rd^ leg (arrows indicate styli). Scale bars: 100 μm (**A**); 20 μm (**B–I**).

**Figure 2.**
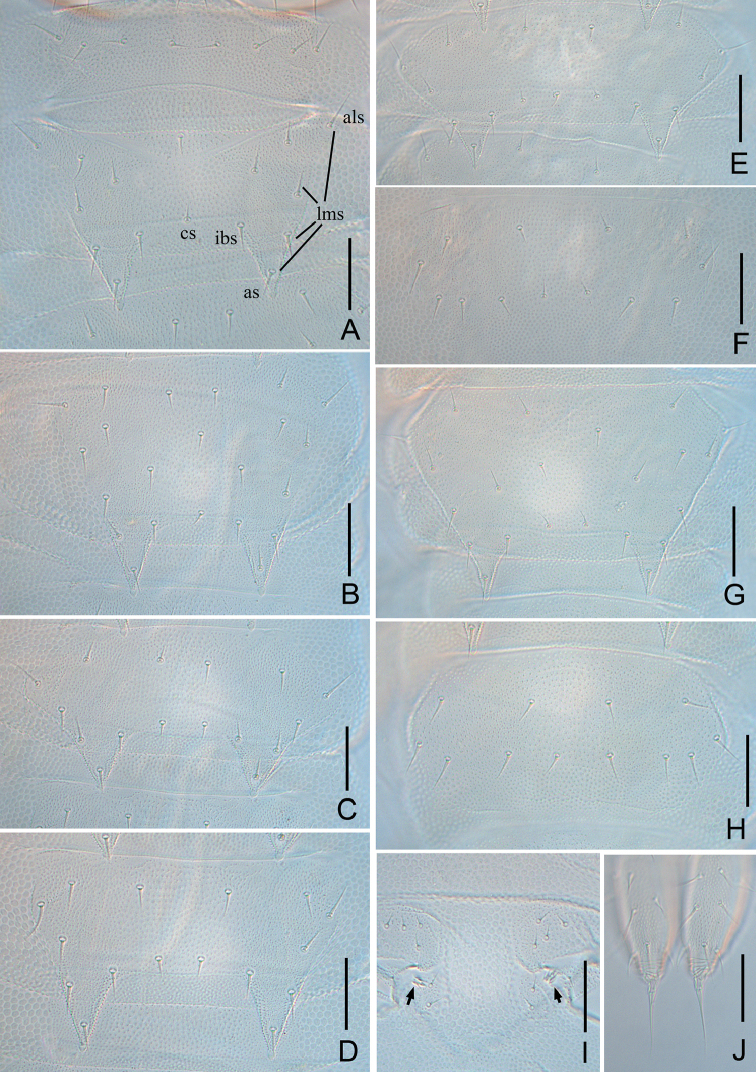
*Symphylella minuta* sp. nov. **A** 1^st^ and 2^nd^ tergites (als-anterolateral seta, as-apical seta, cs-central seta, ibs-inner basal seta, lms-lateromarginal seta) **B** 3^rd^ tergite **C** 4^th^ tergite **D** 5^th^ tergite **E** 13^th^ tergite **F** 14^th^ tergite **G** 15^th^ tergite **H** 16^th^ tergite **I** styli and base of 4^th^ leg (arrows indicate styli) **J** cerci, dorsal view. Scale bars: 20 μm.

**Figure 3.**
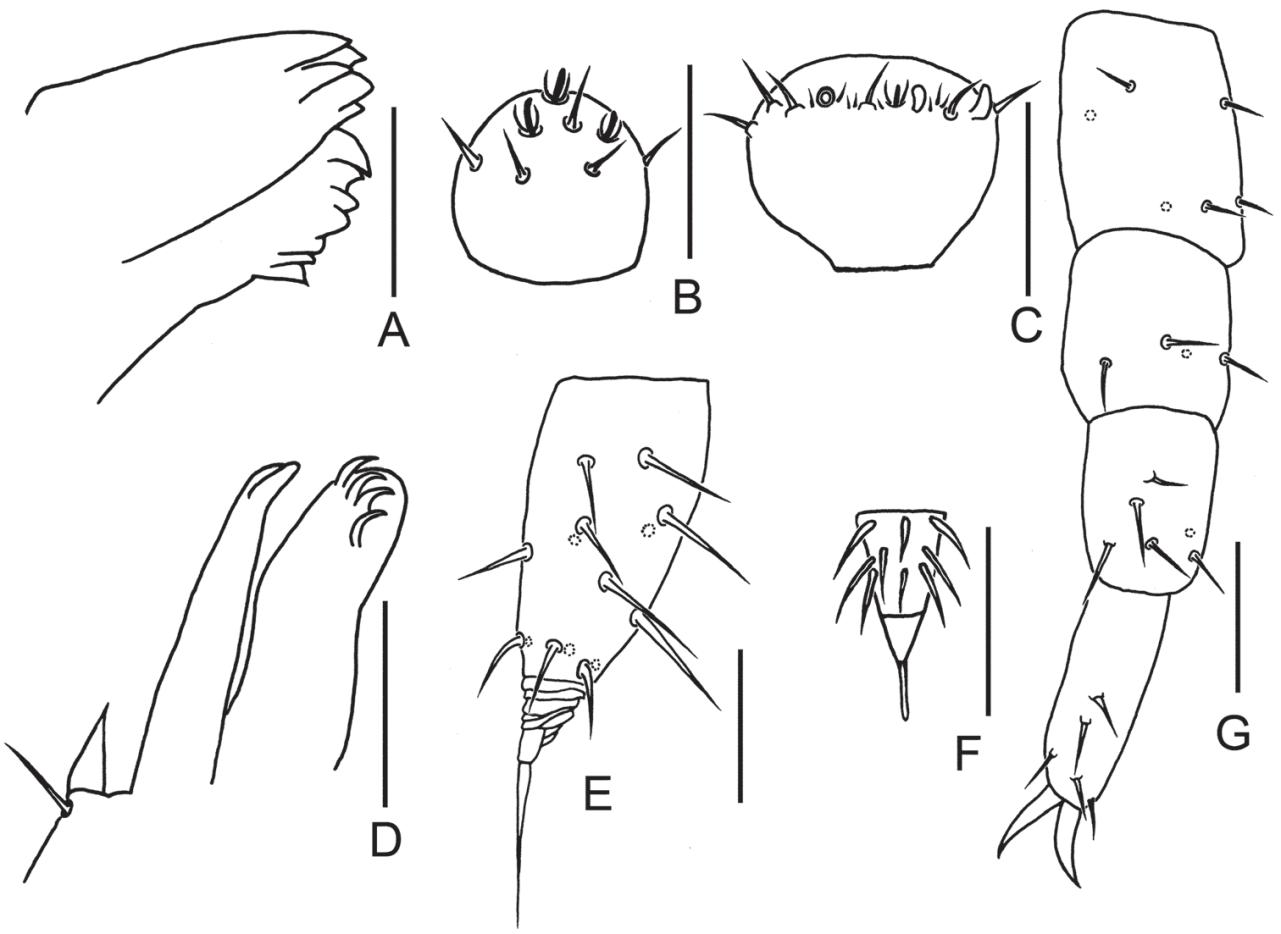
*Symphylella minuta* sp. nov. **A** mandible **B** right apical antennomere, dorsal view **C** right 14^th^ antennomere, dorsal view **D** first maxilla **E** right cercus, dorsal view **F** stylus on base of 4^th^ leg, right side **G** 12^th^ leg. Scale bars: 20 μm (**A–E, G**); 5 μm (**F**).

Figures 1 , 2 , 3 , Tables 1 , 2 , 3

##### Diagnosis.

*Symphylella minuta* sp. nov. is characterized by the delicate and minute body, well-developed but thin central rod with a vestige of a transverse suture in the middle, eight setae on first tergite, pointed processes on tergites, and short cerci with sparse setae.

##### Material examined.

***Holotype***, female (slide no. JS-WX-SY2017020) (SNHM), China, Jiangsu Province, Wuxi City, Daji Mountain, extracted from soil samples of broad-leaf forest, alt. 5 m, 31°32'N, 120°12'E, 9-X-2017, coll. Y. Bu. ***Paratypes***, 1 female (slide no. JS-WX-SY2017031) (SNHM), same data as holotype; 2 females (slides no. JS-WX-SY2020001, JS-WX-SY2020002) (SNHM), ibidem, 14-VII-2020, coll. Y. Bu; 3 females (slides no. SH-DJS-SY2015112, SH-DJS-SY2015114) (SNHM), China, Shanghai, Dajinshan Island, extracted from soil samples of broad-leaf forest, alt. 103 m, 30°41'N, 121°26'E, 22-IX-2015, coll. Y. Bu & Y. L. Jin; 3 females (slides no. ZJ-JLS-SY2019001, ZJ-JLS-SY2019002) (SNHM), China, Zhejiang, Lishui City, Suichang County, Jiulongshan National Nature Reserve, extracted from soil samples of broad-leaf forest, alt. 703 m, 28°13'N, 118°31'E, 27-V-2019, coll. Y. Bu & J. Y. Li. 1 female (slide no. ZJ-GTS-SY2012005) (SNHM), China, Zhejiang, Gutian Mountain, extracted from soil samples of broad-leaf forest, alt. 800 m, 29°15'N, 118°06'E, 11-IV-2012, coll. Y. Bu et al.; Non-type specimens: 7 juveniles with 8–10 pairs of legs, China, Shanghai, Dajinshan Island, extracted from soil samples of broad-leaf forest, alt. 103 m, 30°41'N, 121°26'E, 22-IX-2015, coll. Y. Bu & Y. L. Jin; 1 juvenile with 10 pairs of legs, China, Zhejiang, Lishui City, Suichang County, Jiulongshan National Nature Reserve, extracted from soil samples of broad-leaf forest, alt. 703 m, 28°13'N, 118°31'E, 27-V-2019, coll. Y. Bu & J. Y. Li; 3 juveniles with 9–10 pairs of legs, China, Zhejiang, Gutian Mountain, extracted from soil samples of broad-leaf forest, alt. 800 m, 29°15'N, 118°06'E, 11-IV-2012, coll. Y. Bu et al.; 1 juvenile with 10 pairs of legs, ibidem, 24-IV-2013; 1 juvenile with 10 pairs of legs, ibidem, 16-V-2012; 1 juvenile with 9 pairs of legs, ibidem, 19-VI-2012; 1 juvenile with 7 pairs of legs, ibidem, 15-VII-2012; 2 juveniles with 7 and 9 pairs of legs, ibidem, 14-X-2012; 2 juveniles with 9–10 pairs of legs, ibidem, 17-XI-2012, coll. Y. Bu et al.

##### Description.

Adult body 1.5 mm long in average (1.2–2.2 mm, *n* = 8), holotype 1.4 mm (Fig. 1A).

***Head*** length 150–170 μm, width 133–158 μm, with widest part somewhat behind the middle on a level with the points of articulation of mandibles. Central rod well developed but thin, with a vestige of a transverse suture in the middle. Anterior branches normally developed, without median branches. Dorsal side of head moderately covered with setae of different length, longest setae (18–25 μm) located most anterior on head, at least 3.0 times as long as central ones (4–6 μm). Cuticle of anterolateral part of head with rather coarse granules, around Tömösváry organ with moderate granules, other area with fine and faint granulation (Fig. 1F).

***Tömösváry organ*** globular, diameter 11–14 μm, about half of greatest diameter of third antennomere (24–27 μm), opening small and round (5–8 μm), with distinct vertical inner striate (Fig. 1F).

***Mouthparts*.** Mandible with two fused lamellae and 12 teeth in total (Fig. 3A). First maxilla has two lobes, inner lobe with four hooked teeth, palp bud-like and pointed (Fig. 3D). Anterior part of second maxilla with many small protuberances each carries one seta, distal setae thicker and spiniform; posterior part with sparse setae. Cuticle of maxilla and labium covered with dense pubescence (Fig. 1G).

***Antennae*** with 14–19 antennomeres (holotype with 19 on left, 14 on right), length 320–380 μm (350 μm in holotype), about 0.2 of body length. First antennomere cylindrical, greatest diameter almost two times wider than long (23–27 μm, 14–16 μm), with three or four setae on inner side, longest inner seta 8–10 μm, outer side without seta. Second antennomere wider (22–29 μm) than long (16–19 μm), with six to eight setae evenly inserted around the antennal wall with interior setae (10–18 μm) slightly longer than exterior ones (6–7 μm). Chaetotaxy of third antennomere similar to preceding ones. Setae on proximal antennomeres longer than on distal ones. Proximal antennomeres with only primary whorl of setae, middle and subapical antennomeres with secondary whorl setae present. Three kinds of sensory organs observed on antenna: rudimentary spined sensory organs on dorsal side of middle antennomeres (Figs 1C, 3C); cavity-shaped organs on antennomeres 5–19 (Figs 1C, 3C); bladder-shaped organs on antennomeres 7–14 next to apical one increasing in number on subapical antennomeres to a maximum of 12 (Figs 1D, E, 3C). Apical antennomere subspherical, somewhat wider than long (width 22 μm, length 13–17 μm), with 10–13 setae on distal half; three or four spined sensory organs consisting of three or four curved spines around a central pillar in depressions on distal surface (Figs 1E, 3B). All antennomeres covered with short pubescence. Chaetotaxy and sensory organs of antennae of holotype are given in Table 1.

**Table 1.** Numbers of setae and sensory organs of antennae (holotype).

Antennomeres	No. of primary whorl setae	No. of secondary whorl setae	Rudimentary spined sensory organs	Cavity-shaped organs on dorsal side	Bladder-shaped organs
1^st^	4				
2^nd^	7				
3^rd^	7				
4^th^	8		1		
5^th^	8				
6^th^	8				
7^th^	8			1	
8^th^	9			1	
9^th^	9			1	
10^th^	9			1	
11^th^	9			1	
12^th^	9			1	
13^th^	8		1	1	
14^th^	11	1	1	1	
15^th^	10	4	1	1	2
16^th^	12	4		1	5
17^th^	11	5		1	12
18^th^		5		2	9

***Trunk*** with 17 dorsal tergites. Tergites 2–13 and 15 each with one pair of sharp triangular processes. Length from base to tip of processes distinctly longer than its basal width except for the tergites 4, 7, 10 and 13, in which processes are almost as broad as long; basal distance between processes of tergites distinctly longer than their length from base to tip (Table 2). All processes with end-swellings. Anterolateral setae on all tergites longer than other lateromarginal setae, about 0.6–0.7 of the length of processes on same tergite. Inserted setae (setae between inner basal seta and apical setae) absent. All tergites pubescent (Fig. 2A–H).

**Table 2.** Chaetotaxy of tergites (holotype in brackets).

No. of tergites	lateromarginal	Central setae	Other setae
1^st^	8*		
2^nd^	4 (4)	1 (1)	4 (4)
3^rd^	4–5 (5)	1–2 (1)	9–10 (9)
4^th^	4 (4)	1–3 (2)	4–9 (4)
5^th^	4 (4)	2 (2)	4–5 (4)
6^th^	4–5 (5)	2–3 (2)	10–12 (10)
7^th^	4 (4)	2–3 (2)	4–5 (4)
8^th^	4 (4)	2 (2)	4–6 (4)
9^th^	5 (5)	2 (2)	10–12 (12)
10^th^	4 (4)	2–3 (2)	4–5 (4)
11^th^	4 (4)	2 (2)	4 (4)
12^th^	5 (5)	2 (2)	8–15 (14)
13^th^	4 (4)	2–3 (3)	4 (4)
14^th^			5–14 (5)
15^th^	4–5 (5)	1–2 (2)	6–10 (10)
16^th^			4–13 (4)
17^th^			6–17 (6)

* With 8 setae in a row.

***Tergites*.** Tergite 1 reduced, with eight short setae in a row. Tergite 2 complete, with two triangular posterior processes, four lateromarginal setae, one central seta, anterolateral setae 0.6–0.7 of length of process, longer than other lateromarginal ones, processes 1.1 or 1.2 times as long as broad, basal distance between processes 1.2–1.7 times as long as their length (Fig. 2A). Tergite 3 complete, broader and longer than preceding one with ratios of 0.6–0.8, 1.2–2, and 1.2–1.5 respectively, four or five lateromarginal setae (Fig. 2B). Tergite 4 broader than tergite 3, with ratios 0.7–0.9, 0.9–1, and 1.7–2.2 respectively, four lateromarginal setae (Fig. 2C). Chaetotaxy of tergites 5–7, 8–10, and 11–13 similar as tergites 2–4 (Fig. 2D, E). Pattern of alternating tergite lengths of two short tergites followed by a long tergite only disrupted at the caudal end (Table 2). Tergites 14 and 16 without processes, with 5–14 and 4–13 setae respectively (Fig. 2F, H). Tergite 17 with 6–17 setae. Chaetotaxy and measurements of tergites are given in Tables 2, 3.

**Table 3.** Measurements of tergites and processes (mean ± se, in μm, *n* = 6) (holotype in brackets).

No. of tergites	Length	Width	Length of processes	Basal width of processes	Basal distance between processes
1^st^	24 ± 3 (20)	96 ± 8 (108)			
2^nd^	37 ± 2 (40)	100 ± 6 (108)	21 ± 2 (23)	18 ± 2 (21)	31 ± 3 (28)
3^rd^	64 ± 5 (70)	106 ± 1 (108)	22 ± 1 (24)	16 ± 4 (20)	30 ± 3 (30)
4^th^	39 ± 5 (43)	114 ± 5 (120)	20 ± 2 (22)	22 ± 3 (25)	39 ± 1 (37)
5^th^	40 ± 2 (43)	103 ± 4 (108)	25 ± 3 (25)	20 ± 1 (21)	42 ± 1 (43)
6^th^	77 ± 6 (85)	135 ± 11 (135)	27 ± 2 (30)	21 ± 3 (26)	42 ± 4 (40)
7^th^	43 ± 4 (45)	136 ± 11 (150)	24 ± 2 (26)	24 ± 2 (27)	49 ± 4 (50)
8^th^	45 ± 4 (50)	118 ± 11 (135)	25 ± 4 (31)	21 ± 2 (24)	49 ± 4 (50)
9^th^	79 ± 8 (85)	154 ± 10 (155)	28 ± 2 (31)	20 ± 3 (24)	48 ± 6 (55)
10^th^	44 ± 6 (45)	148 ± 19 (160)	24 ± 2 (26)	90 ± 3 (27)	52 ± 3 (55)
11^th^	36 ± 5 (38)	120 ± 4 (125)	24 ± 4 (29)	20 ± 2 (22)	53 ± 1 (53)
12^th^	76 ± 8 (85)	141 ± 6 (150)	24 ± 1 (24)	21 ± 5 (19)	51 ± 5 (58)
13^th^	45 ± 4 (50)	136 ± 13 (155)	23 ± 3 (27)	22 ± 3 (27)	54 ± 4 (59)
14^th^	47 ± 5 (50)	125 ± 24 (160)			
15^th^	65 ± 7 (75)	135 ± 11 (150)	22 ± 2 (25)	20 ± 3 (22)	44 ± 8 (53)
16^th^	53 ± 7 (63)	113 ± 8 (125)			
17^th^	74 ± 3 (75)	81 ± 7 (75)			

***Legs.*** First pair of legs reduced to two small hairy cupules, each with two long setae (Fig. 1H). Basal areas of legs 2–12 each with four to six setae (Figs 1H, I, 2I). Leg 12 about 0.1 of the body (Fig. 3G). Trochanter 1.1–1.7 times longer than wide (30–46 μm, 24–35 μm), with six subequal setae totally. Femur almost as long as wide (19–25 μm, 20–23 μm), with four setae, three dorsal ones (9–12 μm) distinctly longer than ventral one (5–6 μm); femur pubescent dorsally, laterally with cuticular thickenings in pattern of scales. Tibia nearly 1.2–2.3 times longer than wide (20–26 μm, 11–18 μm), with four or five dorsal setae and one or two ventral setae, longest one 10–12 μm. Tarsus subcylindrical, 2.3–3.0 times as long as wide (16–30 μm, 10–12 μm) with four dorsal setae: two straight and protruding, two slightly curved and depressed; longest setae (20–22 μm) about same length of greatest width of podomere; one to three ventral setae close to claw and distinctly shorter than dorsal ones. Claws curved, anterior one somewhat longer than posterior one, the latter more curved than the former. All legs covered with dense pubescences except areas with cuticular thickenings.

***Coxal sacs*** present at bases of legs 3–9, fully developed, each with three to five setae on surface (Fig. 1I). Relevant area of leg 2, 11 and 12 replaced by two, two or three, and one to three setae respectively (Figs 1H, 2I).

***Styli*** present at base of legs 3–12, subconical (length 2–4 μm, width 2–3 μm), basal part with straight hairs; with a distal blunt apex (2–3 μm) (Figs 1I, 2I, 3F).

***Sense calicles*** with smooth margin to pit, about same length as outer diameter. Sensory seta inserted in cup center, extremely long (90–100 μm).

***Cerci*** short and stout (Figs 1A, 2J, 3E), about one-third of head and one-half of length of leg 12, 2.5 times as long as its greatest width (48–55 μm, 20–22 μm), inserted with sparse subequal setae, six to eight dorsal, five or six ventral, one or two outer, most setae straight, a whorl of six distal setae slightly curved, longest outer seta (14–16 μm) slightly longer than greatest width of cerci, terminal area (8–17 μm) short, 0.3–0.6 time as long as greatest width of cerci, circled by six to eight layers of curved ridges, terminal seta (20–23 μm) long, distinctly longer than terminal area.

##### Etymology.

The species name “*minuta*” refers to the delicate and minute body of the species.

##### Distribution.

China (Jiangsu, Shanghai, Zhejiang).

##### Remarks.

*Symphylella minuta* sp. nov. is similar to *S. oligosetosa* Scheller, 1971 from India and Sri Lanka in the shape of the central rod on head and the shape and chaetotaxy of the tergites. They can be distinguished by the chaetotaxy of the first tergite (eight setae in *S. minuta* sp. nov. vs six in *S. oligosetosa*), shape of the processes (pointed and with swollen ends in *S. minuta* sp. nov. vs strongly pointed and without swollen ends in *S. oligosetosa*), shape and chaetotaxy of cerci (short and stout, about 2.5 times as long as wide in *S. minuta* sp. nov. vs slender, 3.5–3.6 times as long as wide in *S. oligosetosa*). It is also similar to *S. abbreviata* Scheller, 1971 from Sri Lanka in the chaetotaxy of the tergites and shape of the processes, but can be easily separated by the chaetotaxy of the first tergite (with eight setae in *S. minuta* sp. nov. vs six setae in *S. abbreviata*), shape of the third tergite (without indentation in *S. minuta* sp. nov. vs with a distinct middle lateromarginal indentation in *S. abbreviata*), shape of cerci (tapering in *S. minuta* sp. nov. vs with outer side strongly bulging in *S. abbreviata*).

#### Symphylella communa

Taxon classificationAnimaliaScolopendrellidaScolopendrellidae

Jin & Bu sp. nov.

08C107E6-6581-5533-9510-C71111CF490D

http://zoobank.org/3FB07E07-C0D1-47F1-8516-08F61137B7AB

**Figure 4.**
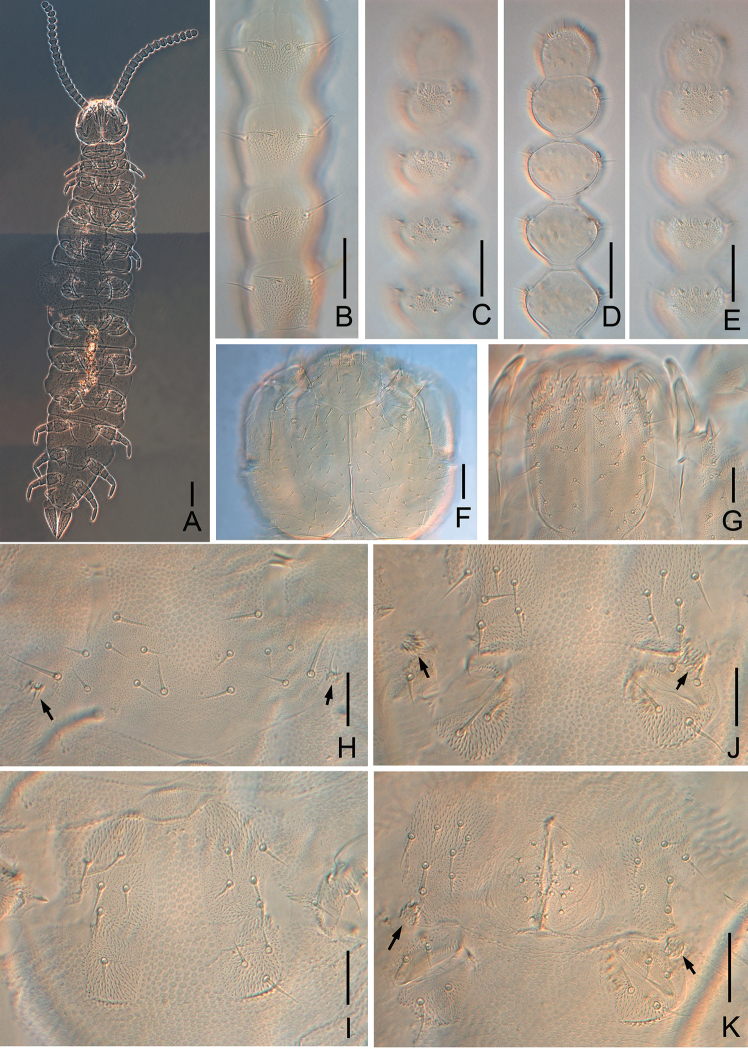
*Symphylella communa* sp. nov. **A** habitus **B** left 2^nd^–5^th^ antennomeres, dorsal view **C** distal five antennomeres, dorsal view **D** distal five antennomeres, show lateral organs **E** distal five antennomeres, ventral view **F** head, dorsal view **G** head, ventral view **H** first pair of legs (arrows indicate the reduced legs) **I** coxa of leg 2 **J** coxa of leg 3 (arrows indicate styli) **K** coxa of leg 4 and male genital plate (arrows indicate styli). Scale bars: 100 μm (**A**); 20 μm (**B–K**).

**Figure 5.**
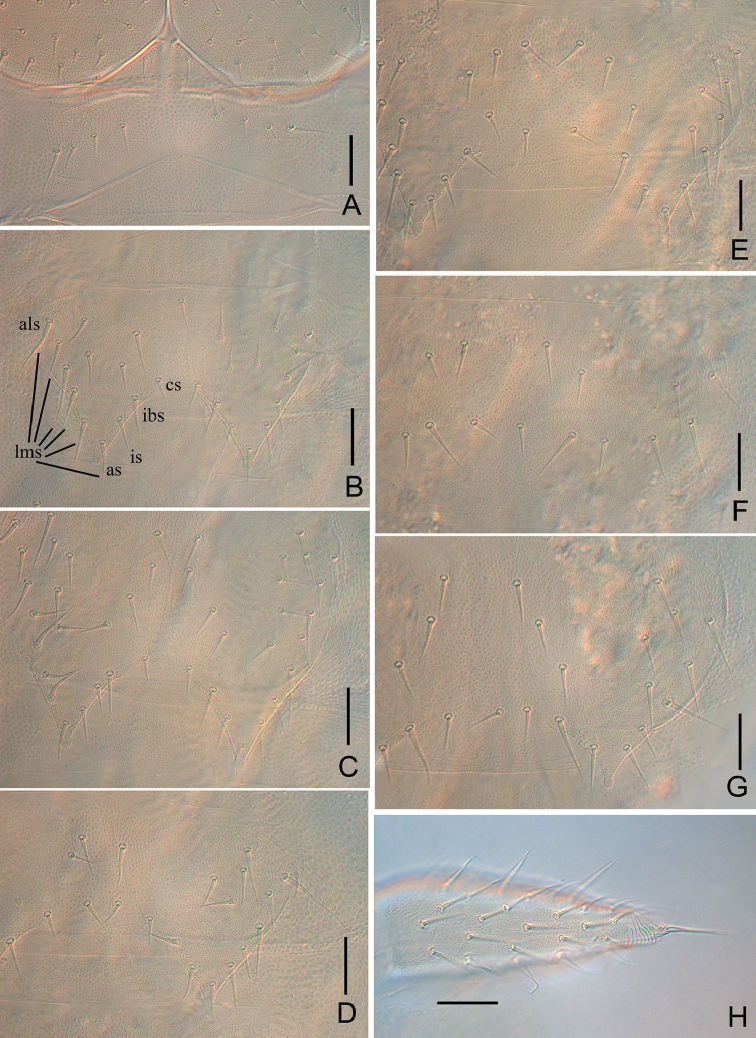
*Symphylella communa* sp. nov. **A** posterior of head and 1^st^ tergite **B** 2^nd^ tergite (als-anterolateral seta, as-apical seta, cs-central seta, is-inserted seta, ibs-inner basal seta, lms-lateromarginal seta) **C** 3^rd^ tergite **D** 4^th^ tergite, right side **E** 5^th^ tergite **F** 14^th^ tergite **G** 15^th^ tergite **H** right cercus, dorsal view. Scale bars: 20 μm.

**Figure 6.**
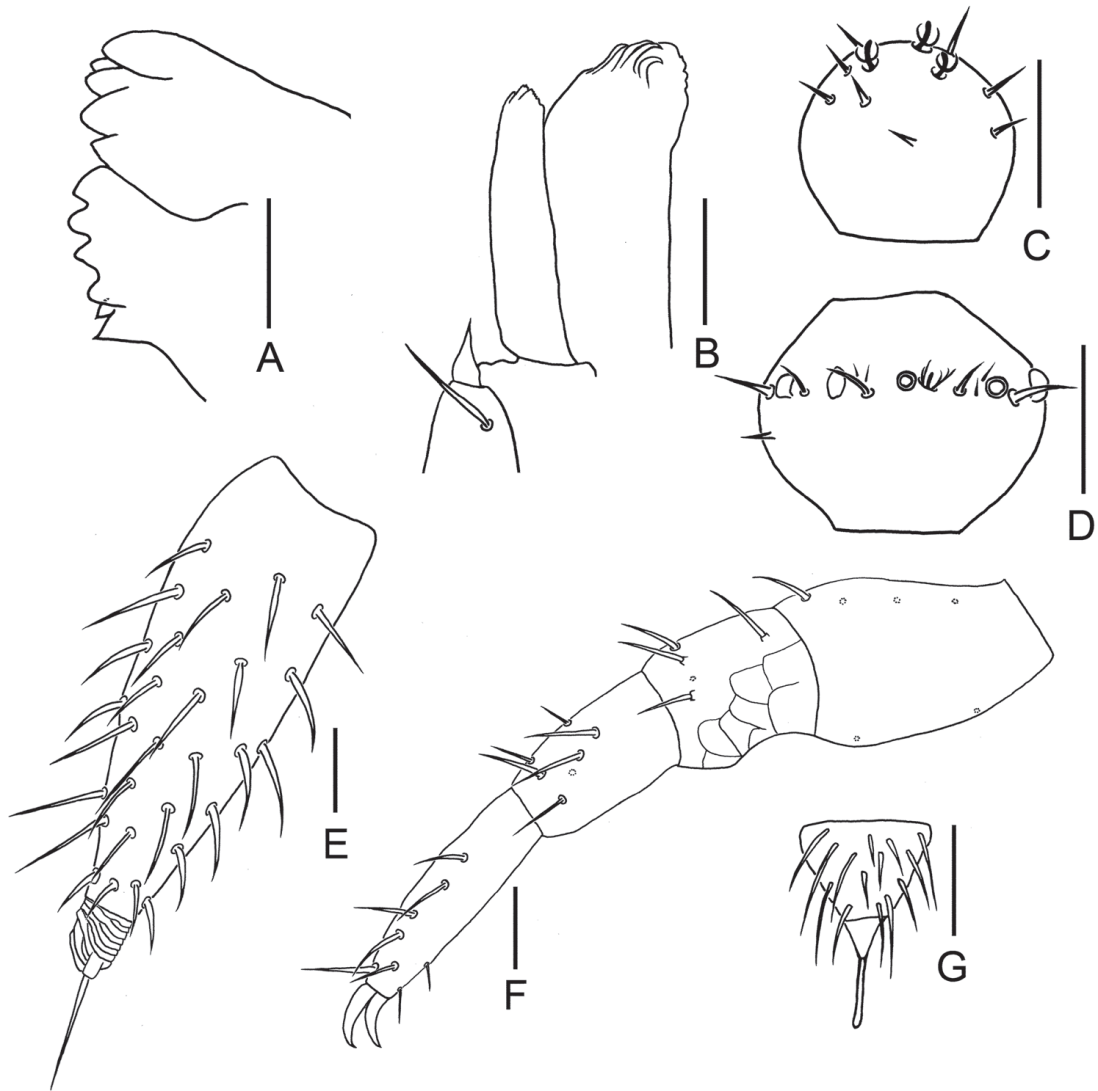
*Symphylella communa* sp. nov. **A** mandible **B** first maxilla **C** left apical antennomere, dorsal view **D** left 13^th^ antennomere, dorsal view **E** left cercus, dorsal view **F** 12^th^ leg **G** stylus on base of 3^rd^ leg, right side. Scale bars: 20 μm (**A–F**); 5 μm (**G**).

Figures 4 , 5 , 6 , Tables 4 , 5 , 6

##### Diagnosis.

*Symphylella communa* sp. nov. is characterized by the chaetotaxy of the first tergite composed by 4+4 setae, processes of tergites somewhat longer or the same length with broad, most of lateralmaginal setae long, anterolateral setae of tergites 2–4, 6, 7, 9, and 10 distinctly longer than other lateromarginal setae, approximately as long as processes of the same tergite, cerci with numerous subequal and slightly curved setae.

##### Material examined.

***Holotype***, male (slide no. SH-DJS-SY2015048) (SNHM), China, Shanghai, Dajinshan Island, extracted from soil samples of broad-leaf forest, alt. 103 m, 30°41'N, 121°26'E, 30-VI-2015, coll. Y. Bu & Y. L. Jin. ***Paratypes***, 1 male (slide no. SH-DJS-SY2015023) (SNHM), 10 females (slides no. SH-DJS-SY2015001, SH-DJS-SY2015018–SH-DJS-SY2015022, SH-DJS-SY2015024, SH-DJS-SY2015025, SH-DJS-SY2015048, SH-DJS-SY2015055) (SNHM), same data as holotype; 2 males (slides no. SH-DJS-SY2015099, SH-DJS-SY2015108) (SNHM), 1 female (slide no. SH-DJS-SY2015107) (SNHM), ibidem, 22-IX-2015; 4 males (slides no. SH-DJS-SY2015083, SH-DJS-SY2015124, SH-DJS-SY2015125) (SNHM), 5 females (slides no. SH-DJS-SY2015058, SH-DJS-SY2015059, SH-DJS-SY2015079, SH-DJS-SY2015122, SH-DJS-SY2015123) (SNHM), ibidem, extracted from soil samples of bamboo forest, 22-IX-2015, coll. Y. Bu & Y. L. Jin; 5 females (slides no. SH-DJS-SY2015117, SH-DJS-SY2015118, SH-DJS-SY2015120, SH-DJS-SY2015121) (SNHM), ibidem, extracted from soil samples of broad-leaf forest, 22-IX-2015, coll. Y. Bu & Y. L. Jin; 1 female (slide no. ZJ-GTS-SY2012018) (SNHM), 1 male (slide no. ZJ-GTS-SY2012009) (SNHM), Zhejiang, Gutian Mountain, extracted from soil samples of broad-leaf forest, alt. 800 m, 29°15'N, 118°06'E, 11-IV-2012, coll. Y. Bu et al.; 1 male (slide no. ZJ-GTS-SY2012021) (SNHM), ibidem, 16-V-2012; 1 female (slide no. ZJ-GTS-SY2012041) (SNHM), ibidem, 19-VI-2012; 1 female (slide no. ZJ-GTS-SY2012045) (SNHM), ibidem, 14-X-2012; 1 female (slide no. ZJ-GTS-SY2012054) (SNHM), ibidem, 17-XI-2012, coll. Y. Bu et al.; 4 females (slides no. JS-WX-SY2017012, JS-WX-SY2017017, JS-WX-SY2017018, JS-WX-SY2017033) (SNHM), 1 male (slide no. JS-WX-SY2017035) (SNHM), China, Jiangsu, Wuxi, Daji Mountain, extracted from soil samples of broad-leaf forest, alt. 5 m, 31°32'N, 120°12'E, 9-X-2017, coll. Y. Bu. Non-type specimens: 37 juveniles with 7–10 pairs of legs, same data as holotype; 6 juveniles with 7–10 pairs of legs, ibidem, 22-IX-2015; 32 juveniles with 7–10 pairs of legs, 22-IX-2015; 8 juveniles with 7–10 pairs of legs, 23-IX-2015, ibidem, extracted from soil samples of bamboo forest, 22-IX-2015, coll. Y. Bu & Y. L. Jin; 1 juvenile with 9 pairs of legs (slide no. ZJ-GTS-SY2012011), Zhejiang, Gutian Mountain, 11-IV-2012; 1 juvenile with 9 pairs of legs, ibidem, 19-VI-2012; 4 juveniles with 8 pairs of legs, ibidem, 15-VII-2012; 1 juvenile with 9 pairs of legs, ibidem, 19-IX-2012; 3 juveniles with 8 or 10 pairs of legs, ibidem, 14-X-2012; 3 juveniles with 8–10 pairs of legs, ibidem, 17-XI-2012; 6 juveniles with 8–9 pairs of legs, ibidem, 12-XII-2012; 1 juvenile with 8 pairs of legs, ibidem, 23-I-2013; 2 juveniles with 8 or 10 pairs of legs, ibidem, 27-III-2013; 1 juvenile with 10 pairs of legs, ibidem, 24-IV-2013, coll. Y. Bu et al.; 12 juveniles with 7–10 pairs of legs, Jiangsu, Wuxi, Daji Mountain, 9-X-2017, coll. Y. Bu.

##### Description.

Adult body 2.2 mm long in average (1.7–3.1 mm, n = 40), holotype 2.1 mm (Fig. 4A).

***Head*** length 200–290 μm, width 200–350 μm, with widest part somewhat behind the middle on a level with the points of articulation of mandibles (Fig. 4F). Central rod distinct, divided into two parts by a node-like interruption in the middle. Anterior branches normal, median branches vestigial. Head dorsally covered with setae of different length, longest setae (25–38 μm) located most anteriorly, at least 3.0 times as long as central shortest ones (8–11 μm). Cuticle on anterolateral part of head with fine coarse granules.

***Tömösváry organ*** globular (Fig. 4F), diameter 13–25 μm, 0.4–0.6 of greatest diameter of third antennomere (34–45 μm), opening round with length about half of greatest diameter of the organ, inner margins of openings covered with uniform vertical striae.

***Mouthparts*.** Mandible with two fused lamellae and 11 teeth in total (Fig. 6A). First maxilla has two lobes, inner lobe with four hooked teeth, outer lobe with teethed apex, palp straight, conical, pointed (Fig. 6B). Anterior part of second maxilla with numerous small protuberances which carry one seta each, distal setae thicker and spiniform; posterior part with sparse setae (Fig. 4G). Cuticle of maxilla and labium covered with dense pubescence (Fig. 4G).

***Antennae*** with 15–20 antennomeres, length 500–720 μm (550 μm in holotype), about one-quarter of body length. First antennomere shorter than second one, greatest diameter 1.2–1.7 times as wide as long (34–43 μm, 20–35 μm), with five to seven setae in one whorl, longest seta (20–21 μm) inserted at inner side and distinctly longer than outer ones (11–12 μm). Second antennomere wider (29–44 μm) than long (23–35 μm), with 6–10 setae evenly inserted around the antennal wall with interior setae (20–22 μm) slightly longer than exterior ones (13–15 μm). Chaetotaxy of third antennomere similar to preceding ones. Setae on proximal antennomeres longer and on distal antennomeres shorter. Proximal antennomeres with only primary whorl of setae (Fig. 4B). Secondary whorl appearing ventrally on antennomeres 6–12 (Fig. 4E). Three kinds of sensory organs on antenna: rudimentary spined sensory organs on dorsal side of most antennomeres except first antennomere (Figs 4D, 6D); cavity-shaped organs on antennomeres 3–6 next to apical one, occasionally on apical antennomere (Figs 4D, 6D); bladder-shaped organs on antennomeres 6–11 next to apical one increasing in number on subdistal antennomeres to a maximum of 16 (Figs 4C, E, 6D). Apical antennomere subspherical, somewhat longer than wide (length 26–38 μm, width 25–28 μm), with 12–18 setae on distal half; two to five spined sensory organs consisting of three or four curved spines around a central pillar in depressions in distal surface (Figs 4D, E, 6C). All antennomeres covered with short pubescence. Chaetotaxy and sensory organs of antennae are given in Table 4.

**Table 4.** Numbers of setae and sensory organs of antennae (holotype).

Antennomeres	No. of primary whorl setae	No. of secondary whorl setae	Rudimentary spined sensory organs	Cavity-shaped organs on dorsal side	Bladder-shaped organs
1^st^	6				
2^nd^	8		1		
3^rd^	9		1		
4^th^	10		1	1	
5^th^	11			1	
6^th^	11			1	
7^th^	11	2		1	
8^th^	11	2	1	1	1
9^th^	12	5		2	2
10^th^	13	6	1	2	3
11^th^	14	6	1	1	2
12^th^	14	5	1		6
13^th^	15	5	1	1	6
14^th^	14	5		2	7
15^th^	13			1	8
16^th^	12		1	1	7
17^th^	12		1	1	4

***Trunk*** with 17 dorsal tergites. Tergites 2–13, and 15 each with one pair of triangular processes. Length from base to tip of processes somewhat shorter than or the same as its basal width; basal distance between processes of tergites 4–13, and 15 longer than their length from base to tip (Table 5). All processes with round swollen ends (Figs 5B–E, G). Anterolateral setae of tergites 2, 3, 4, 6, 7, 9 and 10 distinctly longer than other lateromarginal setae, approach length of process of same tergite (Fig. 5B–D), that of tergites 5, 8, 11, 13 and 15 subequal or slightly shorter than longest ones of other lateromarginal (Fig. 5E, G). Processes with one to three inserted setae. All tergites pubescent (Fig. 5B–G).

**Table 5.** Chaetotaxy of tergites (holotype in brackets).

No. of tergites	lateromarginal	Inserted seta	Central setae	Other setae
1^st^	4+4/5 (4+4)*			
2^nd^	5–7 (6)	1–2 (1)	2–5 (2)	6–14 (8)
3^rd^	7–10 (8)	1–3 (2)	2–5 (2)	14–27 (19)
4^th^	6–8 (6)	1–2 (1)	3–6 (4)	7–18 (11)
5^th^	5–8 (6)	1–3 (2)	2–5 (2)	8–18 (12)
6^th^	8–11 (8)	1–3 (1)	3–6 (5)	21–36 (26)
7^th^	5–7 (6)	0–2 (1)	4–8 (5)	7–20 (15)
8^th^	5–8 (6)	1–3 (2)	3–6 (4)	8–20 (11)
9^th^	7–11 (8)	1–3 (2)	3–6 (4)	21–36 (29)
10^th^	5–7 (6)	0–2 (1)	3–7 (4)	6–19 (11)
11^th^	5–8 (6)	0–3 (1)	3–8 (4)	6–18 (11)
12^th^	6–10 (6)	1–2 (1)	2–6 (4)	9–33 (14)
13^th^	4–7 (5)	0–2 (1)	2–6 (5)	2–15 (7)
14^th^				12–23 (18)
15^th^	5–8 (5)	0–2 (1)	1–4 (3)	5–29 (14)
16^th^				10–16 (14)
17^th^				18–27 (19)

* With 4+4 or 4+5 setae in a row.

***Tergite*.** Tergite 1 reduced, with eight or nine unequal length setae in two groups (4+4 or 4+5) (Fig. 5A). Tergite 2 complete, with two broad posterior processes, five to seven lateromarginal setae, one to three inserted setae, two or three central setae, with anterolateral setae 0.8–1.2 times as long as length of process, processes 0.8–1.0 time as long as broad, basal distance between processes 0.3–0.9 time as long as their length (Fig. 5B). Tergite 3 complete, broader and longer than preceding one with ratios of 0.7–1.3, 0.7–1, and 0.5–1.1 respectively, 7–10 lateromarginal setae (Fig. 5C). Tergite 4 broader than tergite 3, with ratios 1–1.7, 0.8–0.6, and 1–2.6 respectively, six to eight lateromarginal setae (Fig. 5D). Chaetotaxy of tergites 5–7, 8–10, and 11–13 similar as tergites 2–4 (Fig. 5E). Pattern of alternating tergite lengths of two short-tergites followed by a long-tergite only disrupted at the caudal end (Table 5). Tergites 14 and 16 without processes and with 12–26 and 9–16 setae respectively (Fig. 5F). Tergite 17 with 18–27 setae. Chaetotaxy and measurements of tergites are given in Tables 5 and 6.

**Table 6.** Measurements of tergites and processes (mean ± se, in μm, *n* = 22) (holotype in brackets).

No. of tergites	Length	Width	Length of processes	Basal width of processes	Basal distance between processes
1^st^	33 ± 7 (25)	175 ± 25 (150)			
2^nd^	53 ± 4 (58)	135 ± 4 (130)	38 ± 6 (38)	45 ± 7 (43)	23 ± 5 (25)
3^rd^	89 ± 5 (95)	178 ± 10 (168)	41 ± 5 (38)	48 ± 7 (40)	33 ± 5 (40)
4^th^	61 ± 10 (50)	192 ± 12 (178)	36 ± 6 (25)	56 ± 11 (43)	54 ± 9 (65)
5^th^	69 ± 6 (75)	185 ± 13 (180)	43 ± 5 (45)	51 ± 8 (53)	55 ± 11 (48)
6^th^	117 ± 10 (125)	234 ± 12 (225)	49 ± 6 (45)	54 ± 11 (45)	59 ± 11 (53)
7^th^	67 ± 8 (75)	236 ± 13 (232.5)	40 ± 5 (45)	59 ± 10 (63)	79 ± 16 (68)
8^th^	72 ± 4 (75)	194 ± 9 (200)	46 ± 6 (45)	54 ± 10 (50)	70 ± 12 (65)
9^th^	121 ± 4 (120)	258 ± 18 (240)	52 ± 8 (50)	61 ± 13 (50)	63 ± 10 (58)
10^th^	70 ± 9 (75)	239 ± 10 (230)	38 ± 6 (33)	60 ± 15 (55)	81 ± 12 (75)
11^th^	83 ± 8 (75)	219 ± 9 (213)	43 ± 6 (40)	54 ± 11 (43)	73 ± 10 (75)
12^th^	111 ± 10 (100)	258 ± 11 (250)	42 ± 7 (40)	57 ± 14 (50)	68 ± 9 (70)
13^th^	71 ± 4 (70)	233 ± 4 (230)	30 ± 4 (30)	58 ± 9 (50)	72 ± 12 (70)
14^th^	75 ± 13 (70)	198 ± 4 (195)			
15^th^	82 ± 6 (75)	218 ± 14 (208)	31 ± 5 (25)	54 ± 12 (38)	58 ± 9 (58)
16^th^	75 ± 5 (70)	171 ± 7 (163)			
17^th^	111 ± 3 (113)	157 ± 16 (145)			

***Legs*.** First pair of legs reduced to two small, hairy cupules, each with at least one long setae (4–10 μm) (Fig. 4H). Leg 12 about same length of head (Fig. 6F), trochanter 1.2–1.8 times as long as wide (50–90 μm, 36–60 μm), with five to eight subequal setae; femur almost as long as wide (31–53 μm, 30–46 μm), with five or six setae and the dorsally protruding longest setae (20–27 μm) about 0.4–0.7 time of greatest diameter of podomere, laterally with cuticular thickenings in pattern of large scales; tibia nearly 1.3–1.9 times longer than wide (40–65 μm, 23–41 μm), with four to six dorsal setae: three straight and protruding, others slightly curved and depressed, longest seta 0.7–0.9 of greatest diameter of tibia; ventral setae distinctly shorter than dorsal ones; tarsus subcylindrical, about 3.1–4.2 times as long as wide (50–89 μm, 15–22 μm) with six dorsal setae: four straight and protruding, two curved and depressed; longest setae (16–25 μm) about same length or a little longer than greatest width of podomere; one or two ventral setae close to claw distinctly shorter than dorsal ones. Claws curved, anterior one somewhat longer and broader than posterior one, the latter more curved than the former. All legs covered with dense pubescences except areas with cuticular thickenings.

***Coxal sacs*** present at bases of legs 3–9, fully developed, each with four or five setae on surface (Fig. 4J, K). Relevant area of leg 2, 11, and 12 replaced by 1–3, 1–2, and 1–2 setae respectively (Fig. 4I).

***Styli*** present at base of legs 3–12, subconical (length 6–10 μm, width 3–6 μm), basal part with dense straight hairs; distal quarter hairless and blunt (3–5 μm) (Figs 4J, K, 6G).

***Sense calicles*** with smooth margin to pit. Sensory seta inserted in cup center, extremely long (120–180 μm).

***Cerci*** about 0.4–0.8 of head length and leg 12, 2.4–3.3 times as long as its greatest width (115–178 μm, 37–63 μm), moderately covered with subequal length setae (Figs 5H, 6E). Two types of setae inserted on cercus: slightly curved and depressed ones and three or four long and erect outer ones. Longest outer seta (23–32 μm) 0.5–0.7 of greatest width of cerci, terminal area (20–29 μm) short, circled by 7–10 layers of curved ridges. Terminal setae (25–30 μm) 1.0–1.4 times as long as terminal area.

##### Etymology.

The species name “*communa*” is derived from “common” which refer to the common morphology and wide distribution of the species.

##### Distribution.

China (Jiangsu, Shanghai, Zhejiang).

##### Remarks.

*Symphylella communa* sp. nov. is most similar to *S. asiatica* Scheller, 1971 from India and Sri Lanka and *S. macropora* Jin & Bu, 2019 from Tibet in the shape of the central rod, tergites, processes, anterolateral setae, and leg 12. It differs from *S. asiatica* and *S. macropora* in the size of the opening of the Tömösváry organ (moderate in *S. communa* sp. nov. vs very small in *S. asiatica*, extremely large in *S. macropora*) and chaetotaxy of the first tergite (4+4 in *S. communa* sp. nov. and *S. macropora* vs 3+3 setae in *S. asiatica*). It is also affiliated to *S. neotropica* (Hansen, 1903) from Brazil in the shape of tergites and processes, but can be easily distinguished by the central rod (both the anterior and posterior parts are in the same width in *S. communa* sp. nov. vs the anterior part is distinctly narrow than the posterior part in *S. neotropica*).

## Discussion

The genus *Symphylella* is well defined by the presence of 17 tergites with 13 of them having triangular processes present on posterior margins, the first pair of legs replaced by two protuberances with a few setae inserted on, and short styli present on the base of legs 3–9. The shapes of the central rod, Tömösváry organ, tergites and their processes, and cerci, as well as the chaetotaxy of the tergites and cerci, are good characters for the species identification. The measurements of anterolateral setae on tergites are also taxonomically informative for the species definition. The number of lateromarginal setae, inserted setae, central setae, and other setae on tergites often show considerable variations among adult individuals. Asymmetrical setae are usually observed on tergites. In *Symphylella communa* sp. nov., most specimens with 4+4 setae on the first tergite while several specimens exhibits 4+5 and one specimens with 5+5 setae, and the same variation is also observed in *S. macropora*. The shape of the cerci is a relative stable character of the species, but the ratio of the length and width of the cercus may be affected by the mounting process of specimens. Therefore, caution is advised when identify the species of *Symphylella*, which should be based on multiple differential stable characters of fully mature specimens.

The members of the genus *Symphylella* are distributed in almost all faunal regions of the world: 17 species (34.7%) in Nearctic Region, 12 (24.5%) in Ethiopian Region, 11 (22.5%) in Oriental Region, 7 (14.3%) in Palaearctic Region, 6 (12.2%) in Australian Region, and two (4.1%) in Neotropical Region. Most of the species occur in Nearctic, Ethiopian, and Oriental regions. It seems they are more adaptable to the temperate climate. In China, *Symphylella* are often found with high density in the tropical and subtropical area. More species are expected to be discovered in East and South China in the future.

According to the previous studies, the species of the genus *Symphylella* are adapted to diverse habitats and a variety of biotopes: from litter and moss, soil and leaf litter in bamboo and broad-leaf forest, pineapple field, to the plantation of bananas and coco, lawn, botanical garden, tea plantation, orchards, and unoccupied sites. They can live on moist slopes of hills, along streams, in silty soil of agricultural lands, and in caves. They are also present under stone or bricks, in decaying vegetable matter, under bark, in heaps of rotten coconut shells, and under coco trunks on grassy ground (Scheller 1961, 1971). Studies have shown that *Symphylella* species occupy a broad range of habitats, from the littoral intertidal areas to high mountain forests. Some species, *S. asiatica* Scheller, 1971, *S. brincki* Scheller, 1971, *S. foucquei* Scheller, 1971, and *S. tentabundna* Scheller, 1971 for instance, live in mountain areas more than 2000 m above the sea level (Scheller 1971).

*Symphylella* species are supposed to be phytophagous or saprophagous (Rohrbach and Johnson 2003). During our observation of the mounted specimens of *S. communa* sp. nov., we noticed that the intestinal tract of some individuals have many appendages, mandibles, antennae, and cuticle fragments of some other micro-arthropods such as pauropods and collembolans, so this species may be also omnivorous.

## Supplementary Material

XML Treatment for Symphylella

XML Treatment for Symphylella minuta

XML Treatment for Symphylella communa
